# Virus-Induced Membrane Fusion in Neurodegenerative Disorders

**DOI:** 10.3389/fcimb.2022.845580

**Published:** 2022-03-24

**Authors:** Carolina Osorio, Adonis Sfera, Jonathan J. Anton, Karina G. Thomas, Christina V. Andronescu, Erica Li, Rayan W. Yahia, Andrea García Avalos, Zisis Kozlakidis

**Affiliations:** ^1^ Department of Psychiatry, Loma Linda University, Loma Linda, CA, United States; ^2^ Department of Psychiatry, Patton State Hospital, San Bernardino, CA, United States; ^3^ Medical Anthropology – Department of Anthropology, Stanford University, Stanford, CA, United States; ^4^ School of Medicine, University of California, Riverside, Riverside, CA, United States; ^5^ Universidad Nacional Autónoma de México (UNAM), Facultad de Medicina Campus, Ciudad de Mexico, Mexico; ^6^ International Agency for Research on Cancer (IARC), Lyon, France

**Keywords:** fusion, HERVs, cellular senescence, virus, syncytia

## Abstract

A growing body of epidemiological and research data has associated neurotropic viruses with accelerated brain aging and increased risk of neurodegenerative disorders. Many viruses replicate optimally in senescent cells, as they offer a hospitable microenvironment with persistently elevated cytosolic calcium, abundant intracellular iron, and low interferon type I. As cell-cell fusion is a major driver of cellular senescence, many viruses have developed the ability to promote this phenotype by forming syncytia. Cell-cell fusion is associated with immunosuppression mediated by phosphatidylserine externalization that enable viruses to evade host defenses. In hosts, virus-induced immune dysfunction and premature cellular senescence may predispose to neurodegenerative disorders. This concept is supported by novel studies that found postinfectious cognitive dysfunction in several viral illnesses, including human immunodeficiency virus-1, herpes simplex virus-1, and SARS-CoV-2. Virus-induced pathological syncytia may provide a unified framework for conceptualizing neuronal cell cycle reentry, aneuploidy, somatic mosaicism, viral spreading of pathological Tau and elimination of viable synapses and neurons by neurotoxic astrocytes and microglia. In this narrative review, we take a closer look at cell-cell fusion and vesicular merger in the pathogenesis of neurodegenerative disorders. We present a “decentralized” information processing model that conceptualizes neurodegeneration as a systemic illness, triggered by cytoskeletal pathology. We also discuss strategies for reversing cell-cell fusion, including, TMEM16F inhibitors, calcium channel blockers, senolytics, and tubulin stabilizing agents. Finally, going beyond neurodegeneration, we examine the potential benefit of harnessing fusion as a therapeutic strategy in regenerative medicine.

**Graphical Abstract:**
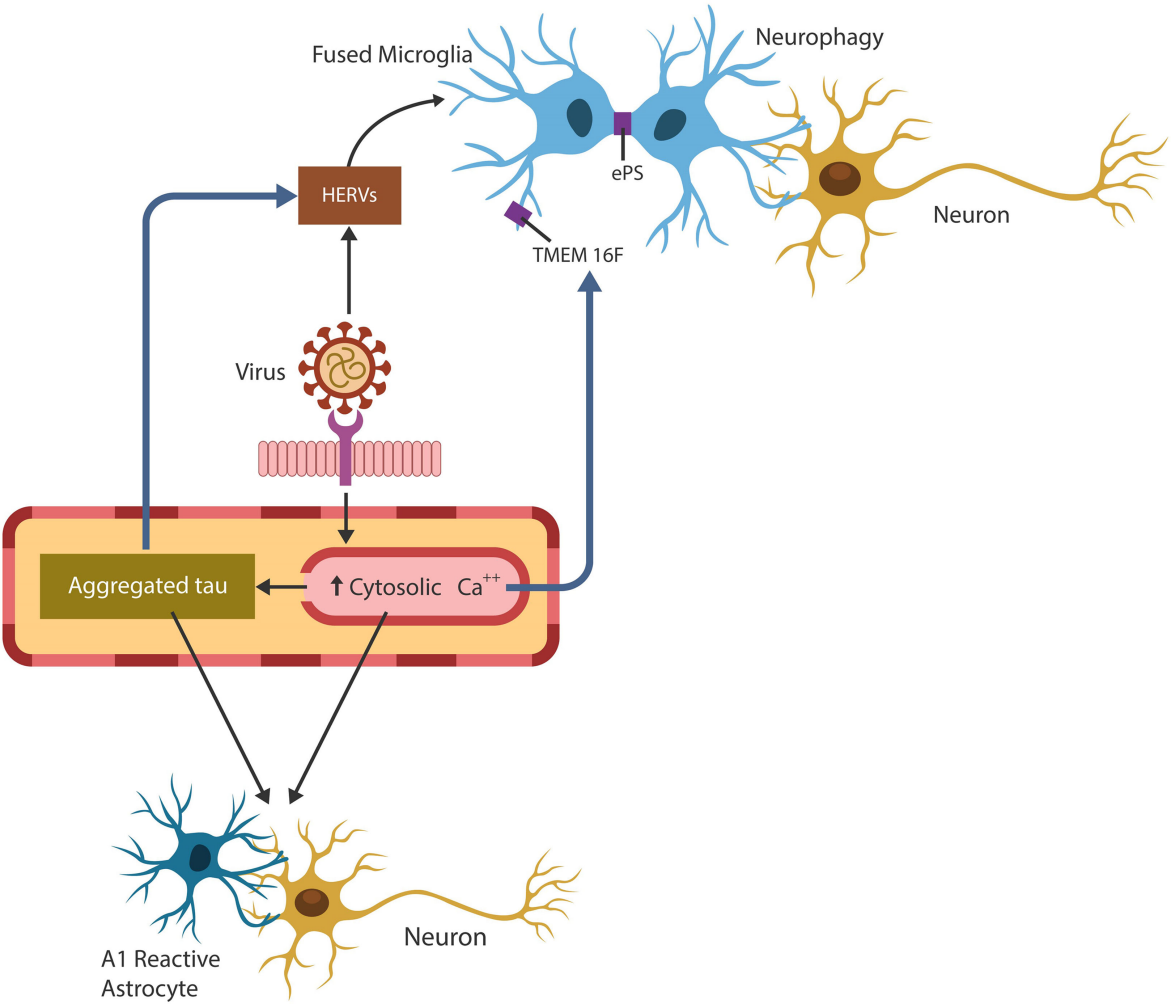
Exogenous viruses hijack human physiological fusogens to generate cellular senescence and immunosuppression, conditions favorable for pathogens’ undetected entry and replication. Pathological cell-cell fusion is initiated by viral arginine motif that drills pores in host plasma cell membranes. The host responds to cellular injury by upregulating cytosolic Ca2+, Tau hyperphosphorylation, TMEM16F activation and phosphatidylserine externalization (ePS). These changes may predispose to neurodegeneration as brain cell-cell fusion results in 1. conversion of supportive to neurotoxic astrocytes, 2. HERVs activation and 3. microglial aberrant phagocytosis of viable neurons (neurophagy) and synapses (synaptophagy).

## Highlights

Enveloped viruses induce cell-cell fusion and syncytia formation to increase infectivity and evade detection.

In hosts, fusion promotes immunosuppression and cellular senescence that may contribute to neurodegeneration by several mechanisms:

1. Viruses spread pathological Tau throughout the extracellular space.
2. Virus-upregulated cytosolic calcium drives Tau hyperphosphorylation and conversion of trophic into neurotoxic astrocytes.
3. Virus-activated HERVs promote microglial fusion and aberrant phagocytosis of healthy synapses and neurons.
4. Neurons with pathological Tau expose phosphatidylserine on the cell surface, facilitating fusion or apoptosis.
5. Viruses may disrupt cognition by altering host cellular cytoskeleton, especially the microtubules.

## Introduction

Cell-cell fusion is a physiological or pathological process in which two or more cells merge their plasma membranes and share the cytoplasm and nuclei, forming syncytia. Under normal circumstances, cell-cell fusion plays a major role in the merger of trophoblasts, gametes, myoblasts, and immune cells (Brukman et al., 2019; Zhang et al., 2020). In addition, physiological syncytia contribute to wound healing and nerve repair (Losick et al., 2013; Neumann et al., 2019). Interestingly, fusogens are derived from viral fossils embedded in human DNA, that can be expressed under pathological circumstances, such as infection with exogenous viruses (Sapir et al., 2008; Balestrieri et al., 2021). In this regard, placental fusogen syncyctin-2 induces immunosuppression necessary for maternal acceptance of the allogeneic fetus, while syncytin-1 triggers placental senescence and immune activation to initiate labor (Cox and Redman, 2017; Gal et al., 2019; Lokossou et al., 2020; Roberts et al., 2021). Viral hijacking of these particular fusogens augments infection by disabling host immune defenses and establishing a virus-friendly environment marked by elevated cytosolic calcium, abundant intracellular iron, and low interferon type I (Martin and Bernard, 2018; Frisch and MacFawn, 2020; Lynch et al., 2021). As cell-cell fusion is a major trigger of cellular senescence, viruses may have developed the ability to exploit host fusogens, promoting premature aging (Gal et al., 2019). Indeed, epidemiological and research data have associated neurotropic viruses with accelerated brain aging and neurodegenerative disorders (Mavrikaki et al., 2021; Sait et al., 2021; Filgueira et al., 2021; Dowd and McKernan, 2021). Along these lines, herpes simplex virus 1 (HSV-1) and human herpesvirus 6 (HHV-6) have been associated with Alzheimer’s disease (AD) and multiple sclerosis (MS) respectively, while human immunodeficiency virus type 1 (HIV-1) contributes to HIV-associated neurocognitive disorders (HAND, 18-19). In addition, cell-cell fusion is associated with the externalization of phosphatidylserine (PS) on the cell surface, a marker of immunosuppression that enables stealthy viral ingress into host cells (Birge et al., 2016). In addition, infection with SARS-CoV-2, the etiologic agent of COVID-19, was accompanied by various cognitive sequelae, linking this virus to neurodegenerative pathology (Paniz-Mondolfi et al., 2020; Wang et al., 2021; Kandemirli et al., 2021; Frontera et al., 2022).

The concept of brain cell syncytia and multinucleation is not new. In the 19^th^ century, Camillo Golgi and Ramón y Cajal were debating whether neurons comprised separate entities or functioned as a brain-wide syncytium (Kiyoshi and Zhou, 2019). In the 20^th^ century, electron microscopy confirmed both viewpoints as under normal circumstances neurons are individual cells, while astrocytes form functional syncytia (Ma et al., 2016). Pathologically however, neurons can fuse with each other or the neighboring cells, forming syncytia. For example, neuron-neuron fusion was documented in aging brains, AD, and MS, linking syncytia to neurodegenerative pathology (Kemp et al.; Hornik et al., 2014). Recent studies in Caenorhabditis elegans (C. elegans) reported fusogen-mediated neuron-neuron and neuron-glia mergers, suggesting that syncytia formation may drive neurocognition (Alexander et al., 2014; Giordano-Santini et al., 2020). Moreover, multinucleated neurons in the supraoptic nucleus were demonstrated in patients with pneumonia, a disease often associated with respiratory viruses, likely implicating virus-usurped host fusogens in this pathology (Ishunina et al., 2000). Fusion of bone marrow cells with Purkinje neurons have been documented by both clinical and preclinical studies, indicating that peripheral and brain cells can merge (Alvarez-Dolado et al., 2003). Furthermore, fused Purkinje neurons were demonstrated in patients with Friedreich’s ataxia, a genetic neurodegenerative movement disorder associated with cellular senescence, calcium (Ca2+), and iron dyshomeostasis (Bolinches-Amorós et al., 2014; Llorens et al., 2019). Interestingly, antiretroviral drug etravirine was found beneficial to patients with Friedreich’s ataxia, perhaps suggesting that the disease may be exacerbated by the activation of human endogenous retroviruses (HERVs) (Alfedi et al., 2019; Lynch et al., 2019).

Tauopathies are neurodegenerative disorders marked by the accumulation of Tau-associated neurofibrillary tangles (NFTs) and cognitive deficits directly correlated with the synaptic and neuronal loss (Giannakopoulos et al., 2003; Dejanovic et al., 2018). Under normal circumstances, the Tau protein is associated with microtubule stabilization and comprises the cellular cytoskeleton of many cell types, including the neurons (Hervy and Bicout, 2019). Pathological Tau (pTau) is hyperphosphorylated and drives neurodegenerative disorders by disrupting both microtubular networks and axonal transport (Millecamps and Julien, 2013; Salvadores et al., 2020).

Many neurotropic viruses, including influenza and SARS-CoV-2, induce cellular senescence and age-related pathology by exploiting the host cellular cytoskeleton and its constituent microtubules (Moujaber et al., 2019; Simpson and Yamauchi, 2020; Wen et al., 2020). Indeed, preclinical studies have found that the envelope (E) of SARS-CoV-1 virus can upregulate intracellular Ca2+ by usurping the host Ca2+ channels in the endoplasmic reticulum Golgi intermediate compartment (ERGIC, 45). As a result, it was suggested that the SARS-CoV-2 spike protein binds Ca2+, facilitating viral infection (Saurav et al., 2021). As Ca2+ is an established regulator of neuronal plasticity, learning, and memory, it is not surprising that Ca2+ dyshomeostasis can promote tauopathies (Vega et al., 2008; Zündorf and Reiser, 2011). Indeed, clinical and preclinical studies have shown that increased intracellular Ca2+ can lead to Tau hyperphosphorylation (Etcheberrigaray et al., 1998; Zempel et al., 2010; Cao et al., 2019). Moreover, recent studies have linked upregulated cytosolic Ca2+ to ferroptosis, an iron-dependent cell death, encountered in AD and other tauopathies (Ashraf and So, 2020; Pedrera et al., 2021; Wang et al., 2022). Although COVID-19 has been associated with hyperinflammatory responses and hypoxia, both of which can upregulate cytosolic Ca2+, this can also be accomplished by virus-induced cellular senescence (Izquierdo et al., 2014; Martin and Bernard, 2018; Danta, 2021; Wicher et al., 2021). In fact, hypoxia may compensate for virus-mediated premature aging, while the accumulation of senescent cells can trigger autoimmune responses (Leontieva et al., 2012; Fukushima et al., 2018; van Vliet et al., 2021).

In this paper, we take the position that virus-induced cellular senescence predisposes to neurodegeneration by upregulating intracellular Ca2+ and iron, increasing ferroptosis and pTau-mediated neuronal loss. In return, this pathology may alter glial homeostasis, contributing to neuronal loss by neurotoxic astrocytes and microglia (Liddelow et al., 2017; Zhang et al., 2020). In this regard, phenomena previously associated with both neurodegeneration and viral infections, including neuronal cycle reentry, aneuploidy, hyperploid DNA, and somatic mosaicism, may be explained by fusion-mediated multinucleation (Knight and Robertson, 2004; Dove et al., 2006; Mosch et al., 2007; Lopes et al., 2009; Arendt, 2012; Miller et al., 2021).

In our previous work, we elaborated on the connection between the SARS-CoV-2 virus and cellular senescence, a subject that will not be discussed in detail here, however, as senescence was associated with microtubular reorganization, the virus may contribute to neurodegeneration by promoting Tau hyperphosphorylation (Ramani et al., 2020; Sfera et al., 2021; Sfera et al., 2021; Pratt et al., 2021) Indeed, pTau, genome destabilization, and HERV activation, may promote neurotoxic astrocytes and microglia, phenomena previously linked to neuronal and synaptic loss (Hornik et al., 2014; Brelstaff et al., 2018; Dittmar et al., 2021; Sokolova et al., 2021). In addition, our focus will be on the cell-cell fusion with less emphasis on virus-host merger, a topic that exceeds the purpose of this review.

In the following sections, we take a closer look at the virus-mediated cell-cell fusion and vesicular merger in the pathogenesis of neurodegenerative disorders. We present a “decentralized” information processing model that conceptualizes neurodegeneration as a systemic illness, triggered by cytoskeletal pathology. We also discuss potential strategies for preventing cellular senescence and immunosuppression, including TMEM16F inhibitors, calcium channel blockers, senolytics, and tubulin stabilizing agents. In addition, we examine the possibility of harnessing fusion as a treatment strategy in regenerative medicine.

## Physiological and Pathological Cell-Cell Fusion

**Figure 1:**
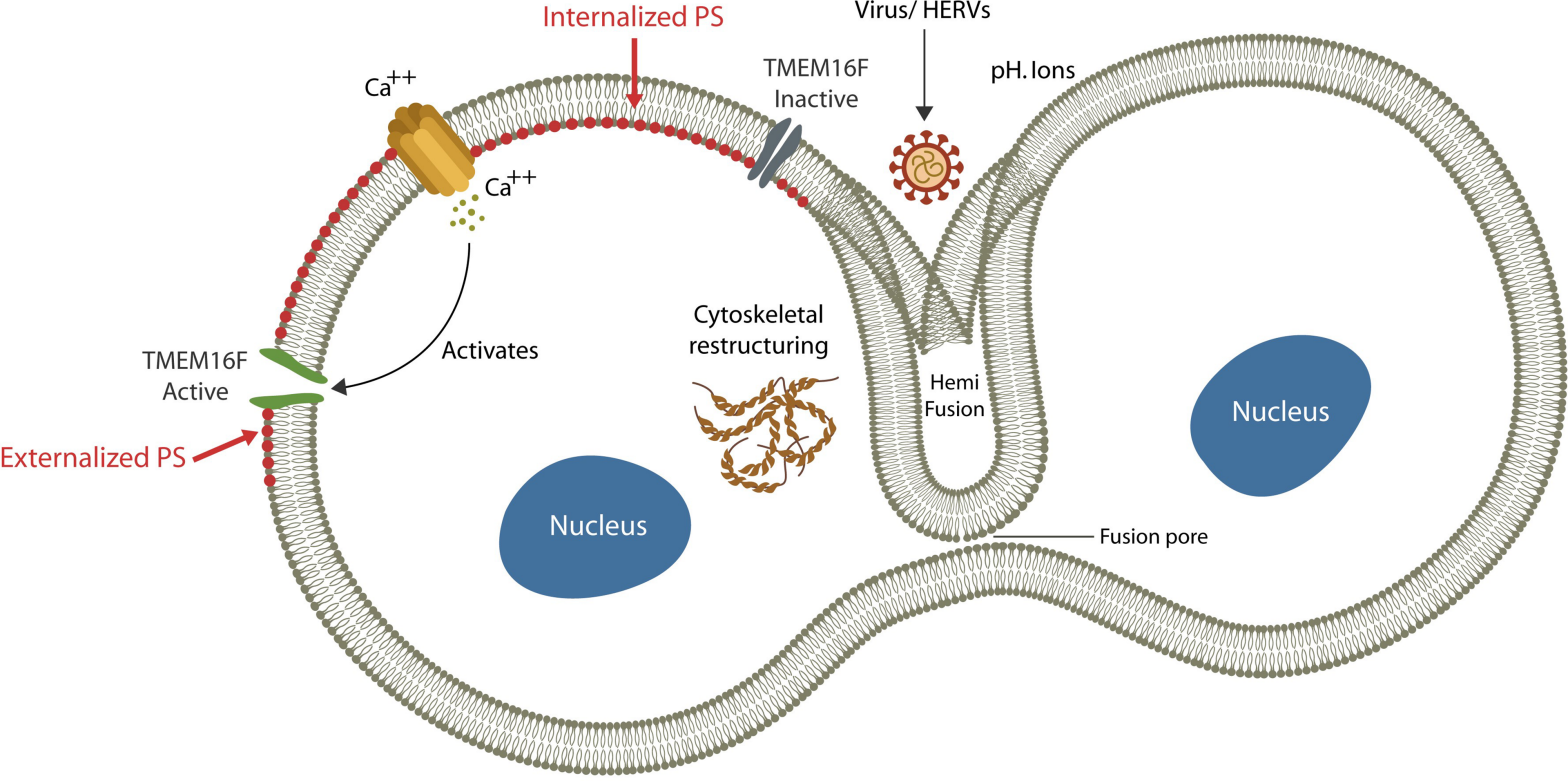
Exogenous viruses and HERVs can hijack physiological fusogens, fusing host cells. The fusion process is comprised of: (1) cytosolic Ca2+ upregulation (*via* extracellular compartment uptake or endoplasmic reticulum release), (2) TMEM16F activation and (3) PS externalization (ePS). Cells undergo fusion or elimination, depending on extracellular pH. Viruses benefit from ePS and elevated cytosolic Ca2+ as the former induces immunosuppression and the later cellular senescence.

Fusion or merging two biological membranes and their lipid bilayers, is a complex process that requires cellular proximity, bridging the outer leaflets of apposing plasma membranes, and the formation of a “stalk”. Subsequently, a hemifusion diaphragm is generated, followed by fusion pore enlargement that ultimately coalesces the two compartments (Akimov et al., 2014) (**Figure 1**). This process requires a shift in the membrane structural asymmetry, including exposing phosphatidylserine (ePS) in the exoplasmic leaflet, a move that triggers immunosuppression (Birge et al., 2016) (**Figure 1**).

Under normal circumstances, the membrane structural asymmetry is maintained by ATP-dependent transporters, including “floppase” (outward transport), “flippase” (inward transport) and “scramblase” (bi-directional transport) that keep phospholipids in their proper leaflet (Pomorski and Menon, 2016). Under normal circumstances PS is localized on the cytoplasmic side and is not usually externalized, except when the cell is damaged and ready for elimination or fusion (Whitlock and Chernomordik, 2021). As both processes are associated with immunosuppression, they facilitate stealthy viral ingress, promoting infection (Birge et al., 2016). Indeed, ePS induces immunosuppression, while upregulated cytosolic Ca2+ is a major driver of cellular senescence (Martin and Bernard, 2018; Zöphel et al., 2020; Wicher et al., 2021). Cells with exposed PS can undergo either fusion or elimination, probably depending on the extracellular pH; an acidic environment promotes fusion, while an alkaline pH favors phagocytosis (Zöphel et al., 2020; Whitlock and Chernomordik, 2021). Indeed, establishing an alkaline extracellular environment is believed to inhibit cell-cell fusion and lower the SARS-CoV-2 infection (Lardner, 2001).

**Figure 2:**
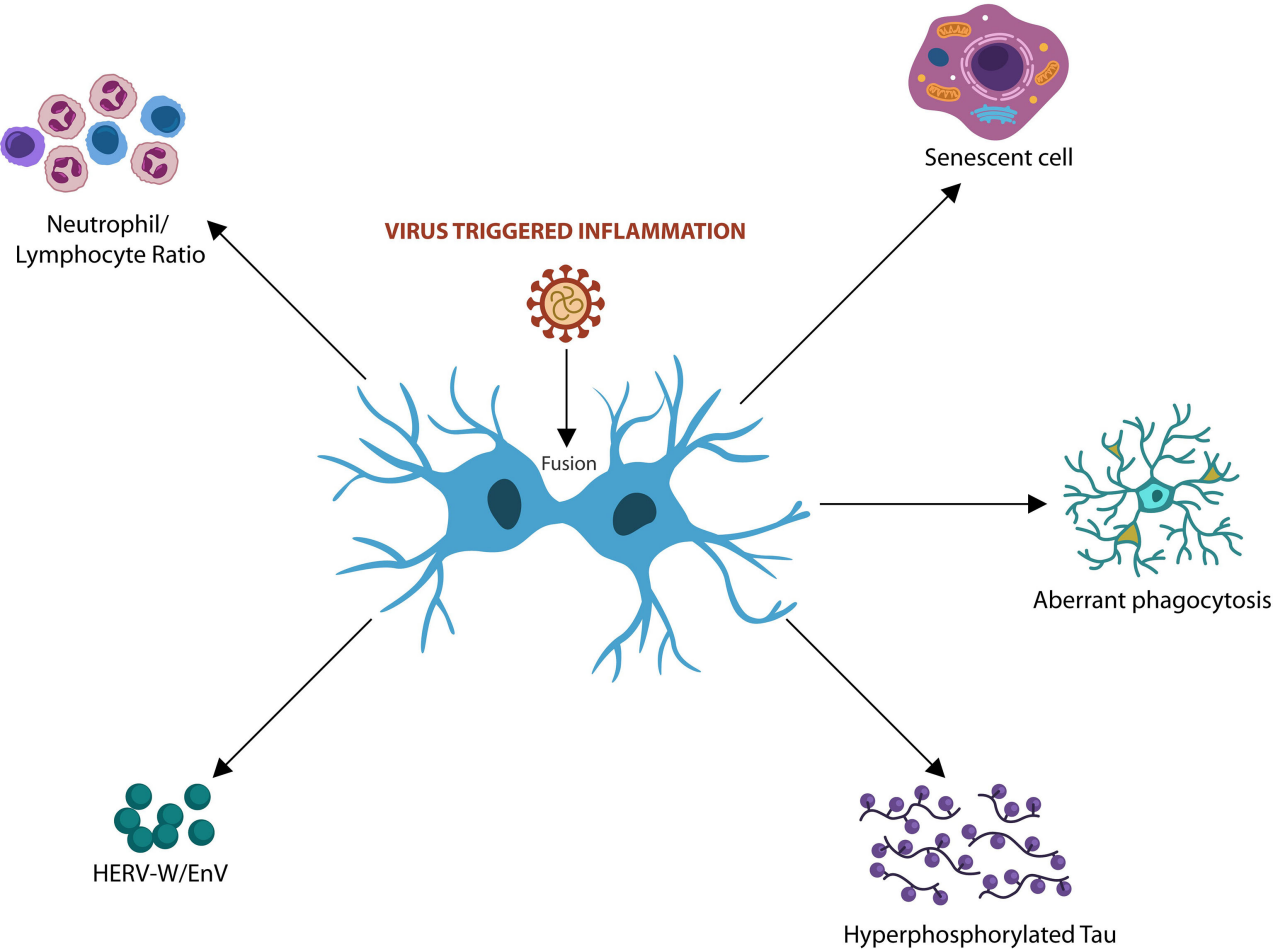
Virus-induced fusion and syncytia formation trigger host cellular senescence and immunosuppression associated with tauopathies. Elevated neutrophil/lymphocyte ratio (NLR) and premature senescence were associated with both viral infections and AD. Virus-activated HERVs and Tau hyperphosphorylation were linked to neurodegeneration. Cell-cell fusion drives aberrant microglia and neurotoxic astrocytes that often engulf viable neurons and synapses, contributing to neurodegeneration.

Placental fusogen syncyctin-2 induces immunosuppression, while syncytin-1, a molecule with superantigen properties, triggers cellular senescence and inflammation, probably to facilitate delivery (Cox and Redman, 2017; Gal et al., 2019; Lokossou et al., 2020; Roberts et al., 2021). By exploiting both fusogens, viruses can manipulate host immunity, inducing immunosuppression and/or immune senescence and exhaustion. At first glance, immunosuppression and cellular senescence appear to be opposites as the former inhibits while the later activates immunity. However, both processes contribute to host “immune failure” as the proinflammatory senescence-associated secretory phenotype (SASP) continuously challenges the immune system into exhaustion. For example, HIV-1 can suppress immunity directly by inducing lymphopenia or indirectly by igniting a senescence-mediated prolonged inflammatory response that causes exhaustion (Appay and Sauce, 2008; Fenwick et al., 2019). Moreover, the SARS-CoV-2 virus was associated with both lymphopenia and inflammation, demonstrating its capability to inhibit host immunity by both mechanisms (Huang and Pranata, 2020) (**Figure 2**).

**Figure 3:**
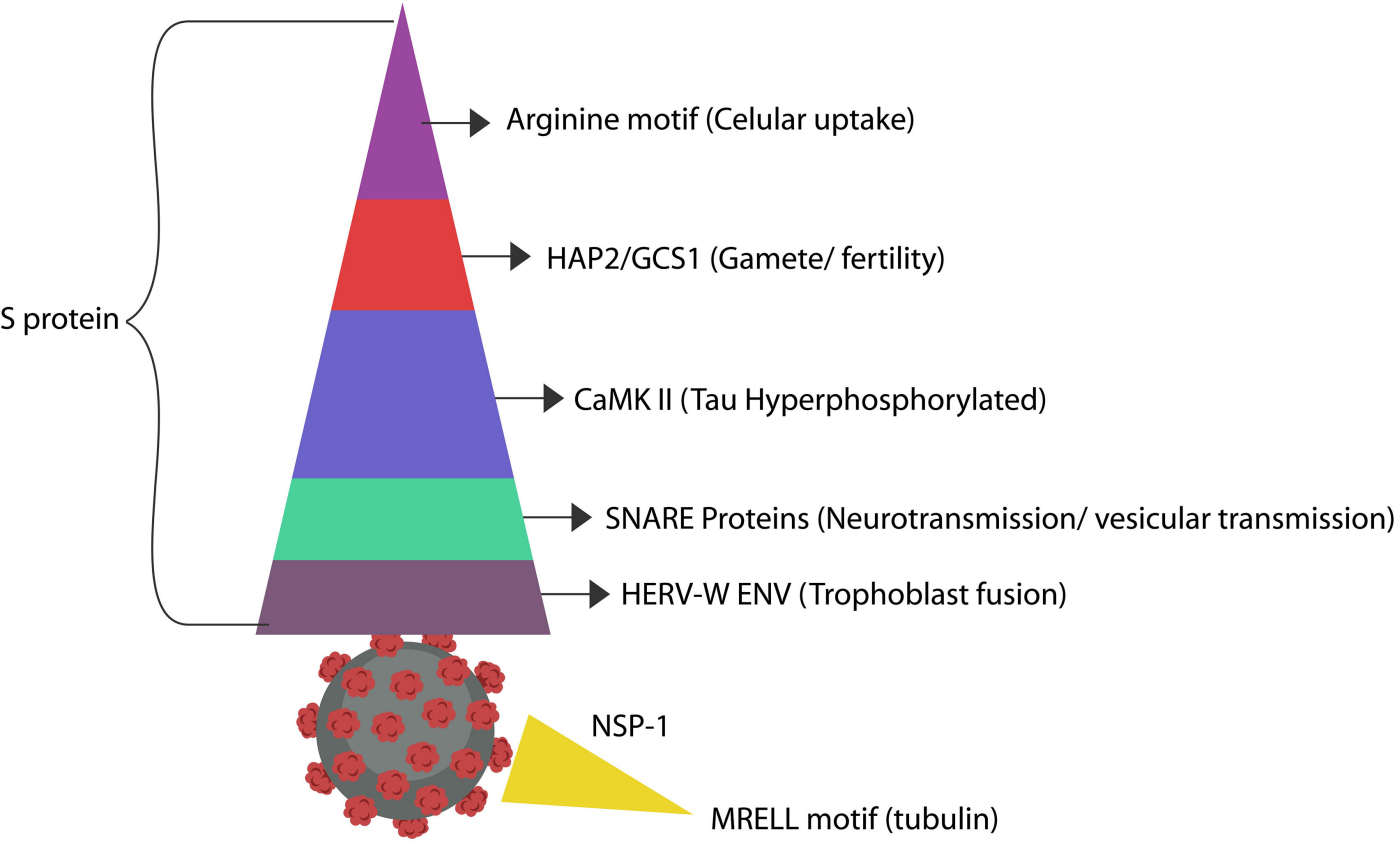
The SARS-CoV-2 antigens display molecular mimicry with numerous host proteins, some of which affect cognition (Yapici-Eser et al., 2021). Arginine and Ca2+ likely convert MAP-Tau to pTau, promoting neurodegeneration. As pTau-containing neurons externalize PS, they may be eliminated by microglia prematurely, outlining another neurodegeneration mechanism (Brelstaff et al., 2018). In addition, pTau perforates cell membranes, likely explaining the mechanism of PS externalization (Lasagna-Reeves et al., 2014). A recent virtual-screening study found that the S protein of the SARS-CoV-2 virus expresses a CaMKII-like system that contributes to the hyperphosphorylation of Tau protein (Wenzhong and Hualan, 2021). However, even in the absence of CaMKII, upregulated cytosolic Ca2+ can promote Tau hyperphosphorylation (Cao et al., 2019). The cysteine-rich motif in the S antigen resembles the extracellular region of HAP2/GCS1, the fusogen involved in gamete merger. SNAP-25 and SNAP-29 are SNARE proteins in charge of organelle fusion and exocytosis. SNAP-25 drives exocytosis in the CNS, while SNAP-29 orchestrates the fusion of autophagosomes with lysosomes (Kádková et al., 2019). These proteins are likely mimicked by the S antigen repeats (Arora et al., 2017; Mastrodonato et al., 2018). In addition, the SARS-CoV-2 protein ORF3a inhibits the fusion of autophagosomes with lysosomes, suggesting molecular mimicry with SNAP-29 protein (Miao et al., 2021). The SARS-CoV-2 S protein was demonstrated to activate HERV-W-ENV gene in T lymphocytes, a protein associated with Alzheimer’s disease (Balestrieri et al., 2021; Licastro and Porcellini, 2021). Another case of molecular mimicry was found between the SARS-CoV-2 NSP-1 antigen and host tubulin, likely connecting this virus to dysfunctional information processing (Sobhy, 2021).

Several viruses, including Zika, promote host immunosuppression by hijacking HAP2/GCS1, a physiological fusogen associated with gamete fusion and zygote formation, disrupting these processes (Polack et al., 2005; Liu et al, 2015; Valansi et al., 2017). In addition, the established fusogenic pathogen, respiratory syncytial virus (RSV), enters host cells *via* a HAP2/GCS1-like cysteine-rich region, lowering host immunity (Bertrand et al., 2013; Fédry et al., 2017). As the S antigen of SARS-CoV-2 virus contains many cysteine-rich repeats, it may be easily recognized by HAP2/GCS1, triggering infertility (Puthenveetil et al., 2021) (**Figure 3** and **Table 2**). Moreover, the HIV-1 trans-activator of transcription (Tat) protein contains cysteine-rich regions, suggesting HAP2/GCS1 exploitation (Bertrand et al., 2013).

Aside from peptides, physiological fusogens are also comprised of amino acids, fusion-associated small transmembrane (FAST) proteins, and chemical agents, including dextran sulfate, Ca2+, and sodium nitrate (Goujon et al., 2015; Abdou and Henderson, 2019; Chan et al., 2021). As opposed to neurons, astrocytes generate physiological syncytia and share their cytoplasm through gap junctions formed by connexin 30 or 43 (Xing et al., 2019). In response to cytosolic Ca2+, astrocytes release gliotransmitters, maintaining network homeostasis (Guerra-Gomes et al., 2018). Connexin 43 (Cx43), implicated in both AD and HAND, likely plays a major role in the pathogenesis of neurodegenerative disorders *via* neurotoxic astrocytosis (Kajiwara et al., 2018). Indeed, novel studies have associated Cx43 with both HIV-1 antigen Tat and pTau, connecting these proteins to HAND and neurodegeneration (Berman et al., 2016; Fuglewicz et al., 2017).

Taken together, exogenous viruses hijack host physiological fusogens to lower antiviral immunity and induce cellular senescence, conditions favorable for viral entry and thriving. In susceptible hosts, these processes may predispose to neurodegenerative disorders by several mechanisms, including pathological Tau dissemination, upregulated cytosolic Ca2+ driving Tau hyperphosphorylation, conversion of trophic into neurotoxic glia, Tau-mediated ePS and cytoskeletal dysfunction.

## Cell-Cell Fusion in Neuropsychiatric Disorders

The connection between intracellular pathogens and neuropsychiatric symptoms has been known for a long time. Malaria, toxoplasmosis, and lately *Porphyromonas gingivalis* have been associated with psychopathology, while several viruses were linked to neurodegeneration and developmental disabilities (Henry et al., 2010; Lima et al., 2021; Olsen, 2021; Chemparthy et al., 2021). Along these lines, the 1918 influenza pandemic and the more recent H5N1 epidemic were associated with Parkinson’s disease and autism (Maurizi, 2010; Shuid et al., 2021). In addition, offspring of mothers pregnant during the 1964 rubella epidemic were more likely to develop autism and schizophrenia in adulthood compared to the general population (Brown et al., 2000). However, the question begging for an answer is: can viruses disrupt host cognition directly?

### Virus-Induced Neurocognitive Dysfunction, Direct Mechanisms

Several modalities of unmediated viral interference with host neurocognitive brain areas were recently described, including pTau dissemination, anti-pTau antibodies, molecular mimicry with cellular cytoskeleton, and direct viral invasion of host cognitive centers.

1. Several viruses, including SARS-CoV-2, were demonstrated to spread pTau throughout the brain extracellular space, directly promoting neurodegenerative pathology (Liu et al., 2021).
2. A recent COVID-19 study found that pTau could elicit autoantibodies, exacerbating end-organ damage, probably including the brain (Magalhães et al., 2021). Along these lines, earlier preclinical studies have reported that tauopathies can be initiated by anti-pTau autoantibodies (Yanamandra et al., 2017).
3. Several viruses mimic host microtubular proteins, altering cognition at the cytoskeletal level. For example, HIV-1 imitates microtubular end-binding protein 1 (EB1), disrupting cognition directly (Naghavi, 2021). Other viruses, including Influenza A and Zika, exploit the host microtubular network, while SARS-CoV-2 may accomplish the same *via* a tubulin-like MREL motif located in the NSP1 protein (Simpson and Yamauchi, 2020; Sobhy, 2021).
4. The SARS-CoV-2 virus can directly invade the brain cognitive centers, inducing Alzheimer’s-like neuropathology (Shen et al., 2022).
5. The S antigen of SARS-CoV-2 may contain a Ca²^+^/calmodulin-dependent protein kinase II (CaMKII) motif that could alter the microtubules directly (Wenzhong and Hualan, 2021)(please see the “*Fusion by Calcium*” section).

### Virus-Induced Neurocognitive Dysfunction, Indirect Mechanisms

Aside from the direct effect of viral proteins on microtubules or their constituent, tubulin, viruses can lead to pTau accumulation indirectly *via*:

1. Cellular senescence
2. HERV activation
3. Cytosolic Ca2+ and iron upregulation,
4. Neurotoxic glia, and
5. Vesicular trafficking (Nieto-Torres et al., 2014; Simpson and Yamauchi, 2020; Wen et al., 2020; Spotorno et al., 2020).

Extra and intracellular vesicular trafficking are discussed below, while the other mechanisms are described in the following sections.

### SNARE Proteins and Vesicular Trafficking

The soluble NSF Attachment Receptor (SNARE) belongs to a superfamily of fusogenic molecules that mediate the merger of intracellular and extracellular vesicles (EVs), including those participating in the formation of immunological synapse (the interface between the T cells and antigen-presenting cells) (Das et al., 2004; Koike and Jahn, 2019). Vesicular fusion machinery is composed of v and t-SNAREs and their components, the synaptosomal associated protein 25 and 29 (SNAP25)(SNAP-29) highly expressed in neurons (Arora et al., 2017; Mastrodonato et al., 2018) (**Figure 3** and **Table 1**).

**Table 1:** Physiological fusogens exploited by endogenous or exogenous viruses *via* molecular mimicry.

PHYSIOLOGICAL FUSOGENS	VIRUS	FUNCTION	REFERENCES
Syncytin-1	Influenza A/HERV-W	Senescence/inflammation/labor	(Moujaber et al., 2019; Simpson and Yamauchi, 2020; Wen et al., 2020)
Syncytin-2	HERV-FRD	Trophoblast fusion/placental exosomes	(Cox and Redman, 2017; Gal et al., 2019; Lokossou et al., 2020; Roberts et al., 2021)
HAP2/GCS1	Zika virus	Gamete fusion	(Valansi et al., 2017)
Arginine	SARS-CoV-2 and Influenza-A	Myoblast/vesicle fusion	(Das et al., 2004; Koike and Jahn, 2019; Gong et al., 2021)
SNAP25/SNAP-29	SARS-CoV-2	Fusion intracellular vesicles	(Yapici-Eser et al., 2021)
Ca2+	SARS-CoV-2/HIV	Cell-cell/vesicle fusion	(Martin and Bernard, 2018; Zöphel et al., 2020; Wicher et al., 2021)
MAP-Tau	Herpes simplex virus/HIV Tat protein	Microtubule stabilization/fusion	(Hibbard and Sandri-Goldin, 1995; Saylor et al., 2016; Itzhaki, 2017)
TMEM16F	SARS-CoV-2	Trophoblast fusion	(Arora et al., 2017; Kajiwara et al., 2018; Mastrodonato et al., 2018)
Tubulin	influenza A virus	Trophoblast fusion/Cx43	(Simpson and Yamauchi, 2020; Sobhy, 2021)

A recent protein-protein interaction (PPI) study reported that the SARS-CoV-2 mimics SNAP25 and SNAP-29, exploiting vesicular transport to egress host cells (Ghosh et al., 2020; Yapici-Eser et al., 2021). These SNARE proteins, characterized by coiled-coil homology domains, facilitate synaptic transmission, memory, and long-term potentiation (LTP), indicating a direct link between viruses and memory (Hou et al., 2006). The SARS-CoV-2 viral protein ORF3a promotes lysosomal exocytosis by inhibiting SNAP-29-mediated fusion of autophagosome and autolysosome (Pan et al., 2005; Barberis et al., 2021; Chen et al., 2021) (**Figure 3**).

## Cell-Cell Fusion and Tau Hyperphosphorylation

Recent studies have associated virus-induced cell-cell fusion with the accumulation and dissemination of pTau throughout the brain extracellular compartment (Miao et al., 2021; Liu et al., 2021). Indeed, exogenous viruses were demonstrated to spread pTau, probably explaining the previously noted prion-like properties of this protein (Lasagna-Reeves et al., 2014; Brunello et al., 2020). Moreover, viruses may exploit pTau for its pore-forming qualities and syncytia formation however, pierced cell membranes may enable extracellular dissemination (d’Errico and Meyer Luehman, 2020). Along these lines, a recent brain organoid study reported abnormal Tau after infection with SARS-CoV-2, suggesting virus-mediated MAP-Tau to pTau conversion (Ramani et al., 2020). Other studies have implicated arginine in the transformation of MAP-Tau into pTau, linking guanidinium side-chains to tauopathies (Walrant et al., 2017). Moreover, virus-mediated Tau hyperphosphorylation was documented in HIV, Influenza A and COVID-19, connecting these viral infections to tauopathies (Brown et al., 2014; Cao et al., 2019).

Recent studies have found that pTau promotes neuronal cell cycle reentry and the subsequent aneuploidy, hyperploidy, and somatic mosaicism documented in both viral infections and neurodegenerative disorders (Knight and Robertson, 2004; Dove et al., 2006; Mosch et al., 2007; Lopes et al., 2009; Arendt, 2012; Miller et al., 2021). Indeed, other novel studies have revealed that pTau can destabilize the genome, activating transposable element (TE), a hallmark of cancer and neurodegeneration (Grundman et al., 2021). In addition, exogenous viruses and pTau can activate HERVs, promoting inflammation and infection (Licastro and Porcellini, 2021). Furthermore, extracellular pTau was demonstrated to alter αV/β1 integrin, converting trophic into neurotoxic astrocytes, emphasizing another neurodegeneration-inducing mechanism (Wang and Ye, 2021).

Aside from its well-established role in neuronal cells, MAP-Tau contributes to the pathophysiology of placenta; it is upregulated in normal pregnancies and lowered in pre-eclampsia (Bergman et al., 2018; Lederer et al., 2020). As viruses disrupt host immunity by converting MAP-Tau to pTau, placental function is likely altered. For example, phosphorylated Tau231, is an early biomarker of pre-eclampsia, linking viruses to reproductive pathology (Brown, 1999; Cheng et al., 2021).

Taken together, viruses promote pTau formation and dissemination to generate cell membrane pores, cell-cell fusion, senescence and immune dysfunction. In this regard, pTau accumulation may predispose to both neurodegeneration and placental pathology.

## Cell-Cell Fusion and Information Processing

Elegant studies in artificially fused unicellular microorganisms demonstrated transfer of learned behavior from one cell to the other, suggesting that rudimentary memory may be stored in the cytoskeletal proteins (Vogel and Dussutour, 2016). In addition, information transfer was detected after fusing two bacteria of different species, indicating that microtubules and tubulin, recently identified in microbes, could participate in this process (Pilhofer et al., 2011; Charubin et al., 2020). Interestingly, human tissues, such as the skeletal muscle, fascia and blood cells may process and store information, further implicating microtubules and tubulin in cognition (Moore and Cao, 2008; Tozzi, 2014; Snijders et al., 2020). In this regard, acquisition of donor personality traits, was documented after cardiac transplants, suggesting that information processing and storing may be a decentralized, blockchain phenomenon (Liester, 2020). Indeed, earlier studies have linked cognition to tubulin and tubulin inhibiting chemotherapy with dysfunctional memory (Craddock et al., 2012; Tuszynski et al., 2020; Kalra et al., 2020). With the same token, treatment with colchicine, a microtubule-disassembling drug, disrupts cognition, further connecting tubulin to information processing (Dent, 2017; Chaldakov, 2018; Sordillo and Sordillo, 2020). Interestingly, tubulin interacts directly with Cx43, the gap junction molecule involved in astrocytic syncytia, suggesting a role in the homeostasis of these cells (Giepmans et al., 2001). Along these lines, tubulin loss in C. elegans was connected to neurodegeneration and dysfunctional neurotransmission (Kraemer et al., 2003).

Recent studies have associated several viruses, including HIV-1, HSV-1, Dengue and Zika, with cognitive dysfunction, suggesting that pathogens can exploit host microtubular networks (Naghavi and Walsh, 2017; Dharan and Campbell, 2018). On the other hand, microtubule-stabilizing agents (MSAs) have demonstrated antiviral and neuroprotective effects, emphasizing a potential therapeutic strategy (Sirakanyan et al., 2021).

Recent studies have shown that microtubules can generate action potential-like electrical oscillations, connecting these proteins to the higher brain functions such as memory and consciousness (Ballatore et al., 2012). Indeed, the recent concepts, “cellular consciousness” and “molecular brains” suggest that cognition and information processing may occur at the cellular level (Baluška et al., 2021; Timsit and Grégoire, 2021). Along these lines, neuronal ribosomal proteins and tubulin were showed to form CNS-like circuits with computation power, indicating the possibility of subcellular information processing (Poirot and Timsit, 2016; Chudinova et al., 2019; Timsit and Bennequin, 2019; Fusco et al., 2021). As viruses usurp host ribosomes to replicate, cognition is likely affected, probably explaining the lethargy of “sickness behavior” observed during viral infection (Li, 2019). Moreover, cytoskeletal proteins were recently linked to inflammation and immunogenicity, suggesting that viruses can induce both immunosuppression and immune activation *via* cytoskeletal manipulation (Moulding et al., 2013; Mostowy and Shenoy, 2015; Tur-Gracia and Martinez-Quiles, 2021). Along these lines, maternal immunological tolerance of the fetus vs. inflammation and preeclampsia may be determined by the viral hijacking of cytoskeleton (Harmon et al., 2016; Wang et al., 2019). Interestingly, the role of the immune system was recently expanded to include information processing and awareness, linking immunity to cognitive processes (Cohen and Efroni, 2019). In addition, proinflammatory cytokines and T cells were found to promote LTP, learning and memory, supporting the concept of immune-based cognition (Bourgognon and Cavanagh, 2020). Indeed, it was hypothesized that “brain superautoantigens”, such as syncytin-1, could drive learning and memory, implicating paleoviruses in human cognitive processes (Nataf, 2017; Mao et al., 2021). Indeed, the viral capsid, activity-regulated cytoskeleton-associated protein Arc, believed to have descended from an ancestral virus, plays a key role in synaptic plasticity, memory and learning (Pastuzyn et al., 2018). Another example, the HERV-K HML-2 (HK2) virus was associated with drug addiction, suggesting that ancient viruses could alter the behavior of modern humans (Karamitros et al., 2018).

## Cell-Cell Fusion, the Molecular Actors

Cell-cell fusion is initiated by the viral arginine motif and its interaction with host cell membrane, Ca2+, TMEM16F, PS, and the cytoskeletal proteins (Whitlock and Chernomordik, 2021) (**Figure 1**). In the following sections, we will introduce the major cell-cell and vesicular fusion players as well as the pathology associated with arginase and arginine, Ca2+ and TMEM16F.

Fusion-induced cellular changes may have body-wide repercussions as they may trigger immunosuppression, cellular senescence, HERV activation, hyperphosphorylated Tau and dysfunctional glia (**Figure 2**).

### Arginase and Nitric Oxide Depletion

Several viruses were found to upregulate host neutrophil/lymphocyte ratio (NLR), by promoting lymphopenia, associated with both COVID-19 critical illness and AD (Kuyumcu et al., 2012; Sayed et al., 2020; Prozan et al., 2021). Elevated NLR likely reflects arginase upregulation and the subsequent depletion of arginine and nitric oxide (NO).

Human neutrophils express high amounts of arginase, therefore upregulation of these cells may cause lymphopenia *via* decreased arginine and NO (García-Navas et al., 2021; Martí i Líndez and Reith, 2021). Indeed, upregulated neutrophils and arginase in HIV-1 infection were shown to deplete arginine that in turn disrupts B and T cell-mediated immunity (Mistry et al., 2001; Munder, 2009; Márquez-Coello et al., 2021). In addition, low arginine-to-ornithine ratio was documented in COVID-19 critical illness, suggesting that the virus hijacks arginase to lower host immunity (Rees et al., 2021). Since older individuals with hypertension and obesity display upregulated arginase, arginine depletion may explain the unfavorable COVID-19 prognosis in this population (Peyton et al., 2018; Moretto et al., 2019). Moreover, the viral arginine motif may mimic the elevated levels of this amino acid, upregulating host arginase by feedback. This mechanism may explain the beneficial effects of arginase inhibitors in both neurodegenerative disorders and viral illnesses (Toque et al., 2013).

Taken together, NLR elevation in severe viral illnesses and AD likely reflects arginase upregulation. Arginase inhibitors may comprise a new therapeutic strategy for both viral illness and neurodegeneration (Ovsepian and O’Leary, 2018). (Please see section “*Arginine: The Fusion Confusion*”).

### Arginine, the Universal “Hole Puncher”

Amino acids are active participants in viral infection and replication and are often exploited by these pathogens (Melano et al., 2021). Arginine, an amino acid with guanidinium side chains, upregulates cytosolic Ca2+ by release from the endoplasmic reticulum (ER) and/or gating through N-methyl-D-aspartate receptor (NMDAR) and glutamate AMPA receptors (AMPAR) (Cunha et al., 2015). Previous studies have established that arginine promotes cell-cell fusion, suggesting that viruses exploit this amino acid for entering host cells (Allolio et al., 2018; Nyenhuis et al., 2021).

Under normal circumstances, arginine functions as a physiological fusogen that facilitates the merger of myoblasts as well as the fusion of intra and extracellular vesicles in the CNS (Das et al., 2004; Koike and Jahn, 2019; Gong et al., 2021). The positively charged guanidinium side chains, a unique characteristic of this amino acid, pierce cell membranes, forming pores that may facilitate both viral uptake and pTau egress (Walrant et al., 2017; Nyenhuis et al., 2021). Along these lines, a novel study linked arginine side chains to pTau, *via* guanidinium π interactions, suggesting that this amino acid plays a major role in tauopathies (Ferrari et al., 2020). Indeed, guanidinium pore-forming properties have helped the development of pharmacological vehicles for intracellular drug delivery, indicating that viruses exploit arginine for its side chains (Wender et al., 2008; Wexselblatt et al., 2014; Trujillo et al., 2015). For example, arginine residues demonstrated in the HIV Tat protein, suggest that viruses hijack guanidinium “keys” to enter host cells (Calnan et al., 1991).

The SARS-CoV-2 virus contains 25 arginine residues, [two in the spike (S) protein and ten in the nucleoprotein (N)], indicating that it is highly fusogenic (Al-Motawa et al., 2020). Indeed, the S protein of SARS-CoV-2 contains a polybasic cleavage motif “Proline-Arginine-Arginine-Alanine (PRRA)” that upregulates host Ca2+ and activates TMEM16F, forming syncytia and lowering lymphocyte levels (Argañaraz et al., 2020; Lin et al., 2021; Zheng et al., 2021). In addition, arginine-induced Ca2+ dysregulation was demonstrated in infection with HSV-1 as well as in tauopathies, linking viruses to neurodegeneration once more (Hibbard and Sandri-Goldin, 1995). Moreover, the SARS-CoV-2 arginine motif activates TMEM16F, a physiological placental fusogen that was implicated in the aberrant glial phagocytosis of neurons and synapses, emphasizing a novel arginine-linked neurodegeneration mechanism (Lin et al., 2021). Furthermore, as arginine-rich peptides were reported to induce cytotoxicity in AD, viral arginine may be the missing link between cell-cell fusion and neurodegenerative disorders (Mamsa and Meloni, 2021).

At the epigenetic level, arginine alters RNA methylation *via* methyltransferase like 14 (METTL14) interaction with N6-methyladenosine (m6A), a mechanism documented in COVID-19 pathogenesis (Li et al., 2021; Zhang et al., 2021). Interestingly, a dysfunctional m6A methylome was recently reported in tauopathies, implicating RNA methylation in these disorders (Jiang et al., 2021).

Taken together, viral arginine plays a major role in hijacking the host fusion machinery, causing immune dysfunction and cellular senescence that likely predispose to neurodegenerative disorders.

### TMEM16F, an Enigmatic Scramblase

The Ca2+ dependent phospholipid scramblase, TMEM16F, alters the structural asymmetry of cell membranes by flipping PS from the cytoplasmic into the exoplasmic leaflet (Younan et al., 2018; Shlomovitz et al., 2019). This marks the cell for either fusion or elimination as microglia can “interpret” ePS as a “fuse me” or “eat me” signal (Whitlock and Chernomordik, 2021). These new findings suggest that the cell-cell merger may protect against aberrant phagocytosis (Kemp et al.; Neher et al., 2012). Indeed, as aging was associated with an increased number of fused neurons, syncytia formation may be neuroprotective by averting the premature elimination of neuronal cells (Kemp et al.; Hornik et al., 2014; Giordano-Santini et al., 2020).

TMEM16F is a physiological fusogen that under normal circumstances mediates the fusion of trophoblasts, indicating that viral overactivation of this protein may trigger placental pathology (Zhang et al., 2020). In the brain, TMEM16F is expressed primarily in neurons and microglia, suggesting that viruses may trigger pathological cell-cell fusion and aberrant microglial behavior (Zhang et al., 2020; Brown, 2021). For example, pathological multinucleated microglia with increased phagocytic capacity were documented in AD, suggesting that these cells can eliminate viable neurons (Kemp et al.; Hornik et al., 2014; Gillispie et al., 2021). Indeed, several neurotropic viruses, including HIV-1 and HSV-1 were demonstrated to fuse microglia, connecting them to neurodegeneration (Borrajo López et al., 2021). In addition, fused microglia were shown to comprise major HIV reservoirs, suggesting that SARS-CoV-2 may also avoid elimination by dwelling in these cells (Wallet et al., 2019). Moreover, as activated microglia can spread pTau and convert trophic to neurotoxic astrocytes, they may contribute to neurodegeneration (Perea et al., 2018; Hopp et al., 2018). Furthermore, as HERV-W ENV, encoding for syncytin-1, induces microglial fusion, it is likely that both exogenous and endogenous viruses may predispose to neurodegenerative pathology (Perron et al., 2005).

### Fusion by Calcium

Increased cytosolic Ca2+ is a major trigger of cellular senescence and many enveloped viruses hijack this ion to generate a replication-friendly environment (Dimitrov et al., 1993; Chen et al., 2019). Ca2+ is a second messenger that under normal circumstances regulates many cellular processes, including fusion, phagocytosis, and vesicular transport as well as the synaptic plasticity in the CNS (Ahluwalia et al., 2001; Nunes and Demaurex, 2010; Mateos-Aparicio and Rodríguez-Moreno, 2020). In the immune system, Ca2+ regulates the immunological synapse, lymphocyte proliferation, differentiation, and apoptosis, suggesting that viral exploitation of Ca2+ signaling can disrupt immunity (Oh-hora and Rao, 2008; Quintana et al., 2011; Pinto et al., 2015). In addition, Ca2+ drives LTP, learning, memory, and information processing *via* its associated proteins, including CaMKII, mitogen-activated protein kinase/extracellular signal-regulated kinase ½ (MAPK/ERKs), and calcium homeostasis modulator 1(CALHM1) (Marambaud et al., 2009).

New studies have shown that CaMKII phosphorylates the virion-like memory protein, Arc, promoting LTP and plasticity, implicating this kinase in neurodegeneration (Zhang et al., 2019). In addition, CaMKII interacts with NMDA receptors in the postsynaptic neurons, further contributing to memory and learning (Lisman et al., 2012). Recent data show that CaMKII can alter T cell responses, indicating that viral hijacking of this protein promotes infection (Trebak and Kinet, 2019). In addition, virus-disrupted Ca2+ homeostasis can lead to synaptic loss, protein misfolding and neurodegeneration (Woods and Padmanabhan, 2012; Popugaeva and Bezprozvanny, 2014; Mazzorana et al., 2016).

Viruses, including SARS-CoV-2, HIV-1 and HSV-1, were shown to usurp Ca2+ and CaMKII, impairing plasticity, learning and memory, linking these pathogens to the neurodegenerative disorders (Gupta et al., 2010; Chen et al., 2020). A new in silico study revealed a CaMKII motif in the S antigen of SARS-CoV-2, suggesting that this virus may disrupt cognition directly (Wenzhong and Hualan, 2021) (**Figure 3** and **Table 2**). Interestingly, CaMKII inhibitors were reported to possess antiviral and antipsychotic properties, further emphasizing the role of Ca2+ signaling in these pathologies (Sałaciak et al., 2021). Ca2+ and tubulin also participates in the functioning of the immune synapse, therefore viral exploitation of this protein may disrupt host immunity (Hui and Upadhyaya, 2017; Sferra et al., 2020).

**Table 2:** Viruses induce immunosuppression and cellular senescence by expressing molecular motifs that mimic physiological fusogens.

Neurotropic viruses	Targets	References
HIV-1 Tat antigen	α- and β-tubulins	(Sayed et al., 2020)
HSV-1	Microtubular network	(García-Navas et al., 2021)
Dengue	Microtubular network	(Martí i Líndez and Reith, 2021)
Zika	β-tubulin	(Munder, 2009)
SARS-CoV-2 MRELL motif	Tubulins/Microtubular network	(Simpson and Yamauchi, 2020)

The cell-cell fusion triggered by these repeats may contribute to pathology, including neurodegeneration.

Taken together, Ca2+ signaling is crucial for the functioning of both the neurological and immunological synapses, indicating that plasticity and immune function are highly intertwined, therefore viral exploitation of the immune system may induce CNS pathology.

## HERVs and Reverse Transcriptase

**Figure 4:**
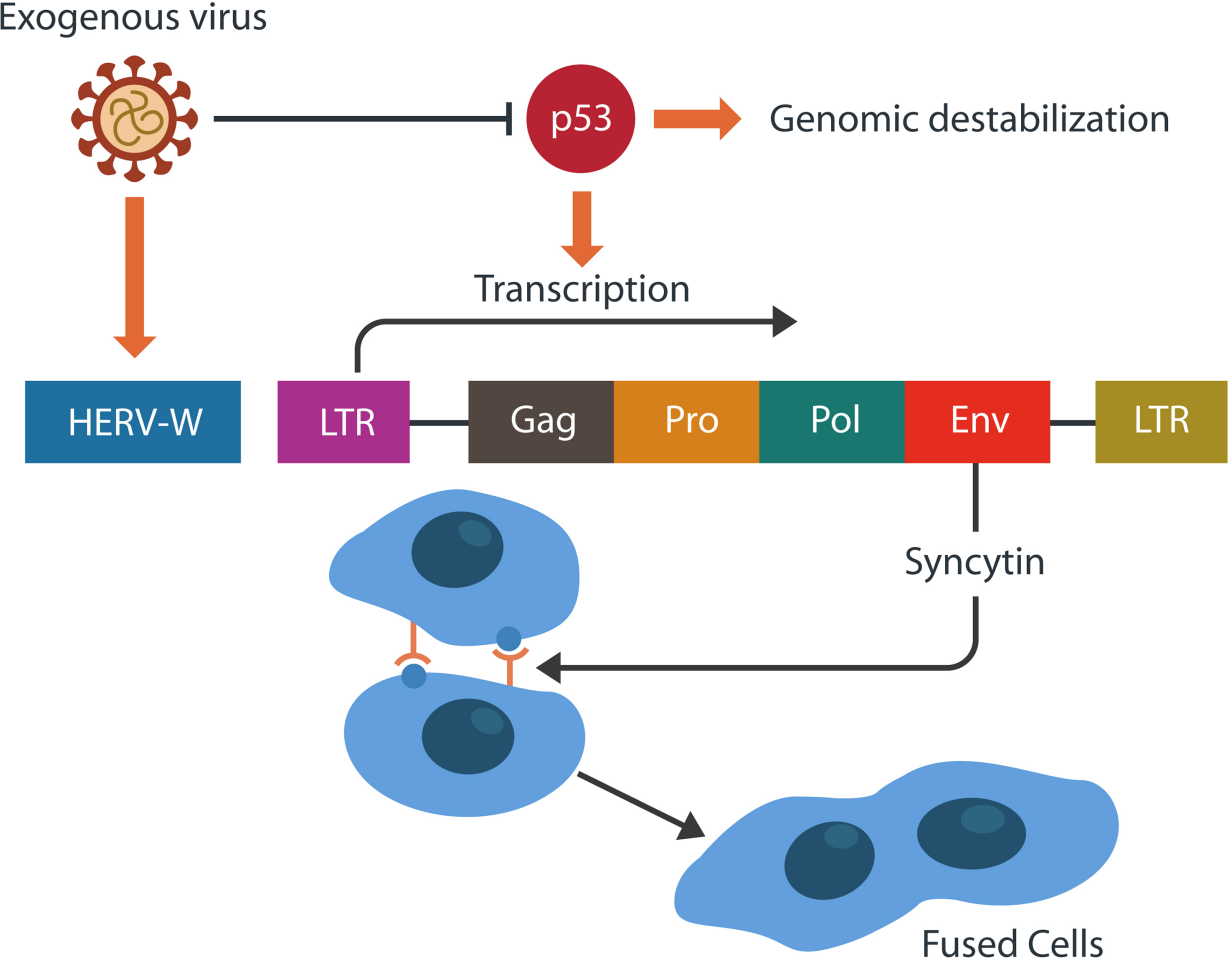
HERVs may retain gag, pol, env genes, and one or two long terminal repeats (LTRs). Env gene encodes for syncytin-1, a physiological placental fusogen. The activity-regulated cytoskeleton-associated protein (Arc) resembles the gag gene and encodes for a retroviral capsid. Viruses often inhibit p53, the tumor suppressor, destabilizing the genome. This in return, facilitates HERV-W transcription and the formation of pathological syncytia.

HERVs comprise about 8% of the human genome that under normal circumstances is epigenetically silenced. At times, some HERV genes may be physiologically or pathologically activated and expressed (Küry et al., 2018). HERVs usually contain one or two long terminal repeats (LTRs), gag (group-specific antigen), pol (polymerase), and env (envelope) genes that encode for proteins, such as syncytin-1 (Mao et al., 2021; Römer, 2021) (**Figure 4**). Age and disease, including exogenous viral infections, may alter genomic methylation, activating the HERV genes (Sultana et al., 2018; Geis and Goff, 2020). Under normal circumstances, HERV-W ENV gene, encoding for syncitin-1, participates in the physiology of placenta but abnormal activation may trigger pathological cell-cell fusion in many organs, including the brain (Dupressoir et al., 2005; Zhuang et al., 2014).

For example, decreased levels of syncytin-1 were associated with pre-eclampsia, while increased expression of this protein was documented in schizophrenia, bipolar disorder and AD (Perron et al., 2013). Indeed, HERV activation plays a major role in several neurodegenerative disorders, including AD, MS, and amyotrophic lateral sclerosis (ALS), suggesting that paleoviruses can trigger contemporary pathology (Cox and Redman, 2017; Lokossou et al., 2020; Roberts et al., 2021; Garcia-Montojo and Nath, 2021).

The SARS-CoV-2 virus was shown to activate HERV-W ENV, suggesting that it could acquire a reverse transcriptase (Zhang et al., 2020; Danta, 2021). Indeed, some COVID-19 patients continue to test positive for viral RNA long time after the recovery, suggesting that SARS-CoV-2 is retrotranscribed or that the virus “hides” in reservoirs, such as microglia and macrophages (Parry et al., 2021). Others have argued against the reverse transcriptase hypothesis, arguing that the SARS-CoV-2 life cycle does not require DNA integration (Yin et al., 2021). However, as the virus can activate HERVs, it may integrate in the genome in tandem with TEs (Jones et al., 2013). For example, SARS-CoV-2 may be hijacked by the Long Interspersed Nuclear Element 1 (LINE-1) retrotransposon and integrated into the DNA without a reverse transcriptase (Singh and Bharara Singh, 2020). Indeed, the Tat antigen of HIV-1 was demonstrated to directly activate LINE-1, indicating that viruses could destabilize the genome (Guo et al., 2018; Zauli et al., 2020; Ramirez et al., 2022).

Several studies found that viruses, including SARS-CoV-2, usurp p53, the tumor suppressor, activating TEs, predisposing to neurodegeneration (Aloi et al., 2015) (**Figure 4**). In addition, preclinical studies have demonstrated pTau-activated TEs, suggesting a different virus-induced neurodegeneration mechanism (Jayadev et al., 2011). Other recent studies connected the loss of p53 to the aberrant microglial phagocytosis, suggesting that damaged DNA can trigger this phenotype, eliminating viable neurons and synapses (Sola et al., 2020; Farmer et al., 2020). Furthermore, as p53 and MAP-Tau maintain genomic stability in tandem, viral manipulation of either protein could activate TEs (Wylie et al., 2016)

A recent preclinical study found that N6-methyladenosine (m6A RNA) can suppress the expression of HERV, suggesting that exogenous viruses may activate HERVs by usurping this epigenetic mechanism (Balestrieri et al., 2021). Indeed, many viruses, including HIV-1, HSV-1 and SARS-CoV-2, were demonstrated to exploit m6A, increasing the risk of TEs mobilization (Tirumuru et al., 2016; Imam et al., 2020; Chelmicki et al., 2021). This is significant as m6A functions as the epigenetic reader of the heterogeneous nuclear ribonucleoprotein A2/B1 (HNRNPA2B1), a molecule disrupted by pTau and implicated in neurodegeneration (Liu et al., 2021; Jiang et al., 2021).

Taken together, HERVs activation by exogenous viruses can cause inflammation as well as p53 and m6A inhibition, predisposing to TE mobilization and neurodegeneration (Dai et al., 2018).

## Harnessing Fusion

Reoviruses are nonenveloped viruses that encode for FAST proteins, inducing cell-cell, but not virus-cell, fusion. For this reason, FAST proteins are excellent tools for harnessing fusion for the treatment of various pathologies, including cancer (Del Papa et al., 2021). In this regard, FAST-containing oncolytic viruses were shown to decrease tumor growth by inducing tumor cell fusion and senescence (Jeon and Jung, 2022). FAST proteins p10, p14, and p15 are promising candidates for cancer gene therapy, but their role in viral infections and neurodegeneration is currently unknown (Del Papa et al., 2021; Brown and Fisher, 2021).

Cell-cell fusion comprises a valuable tool for reprograming fully differentiated human cells into pluripotent ones that can help heal damaged tissues (Pralong et al., 2006; Dörnen et al., 2020). For example, bone marrow-derived stem cells (BMSCs) can restore tissue homeostasis by adopting the properties of those cells (Tan et al., 2021). Regenerative medicine can also repair damaged tissues *via* the highly fusogenic Sendai virus that converts human cells into induced pluripotent stem cells (human iPSCs) (Nakanishi and Otsu, 2012). Moreover, fusion induced with electric pulses or polyethylene glycol (PEG) was demonstrated to promote nerve repair, including the functional restoration of severed axon (Rems et al., 2013; Greenfield, 2018; Neumann et al., 2019).

## Treatment Strategies for Pathological Cell-Cell Fusion

### Arginine: The Fusion Confusion

Viruses hijack arginine for its guanidinium pore-forming properties. The post-fusion upregulation of cytosolic Ca2+ and CaMKII promotes excessive phosphorylation of neuronal nitric oxide synthase (nNOS), increasing NO and peroxynitrite (ONOO−) (Zhou et al., 2016). Although under normal circumstances NO is neuroprotective, peroxynitrite accumulation was associated with neurodegenerative disorders and COVID-19 critical illness (Paris et al., 1998).

Novel SARS-CoV-2 studies have revealed a paradox: both arginine supplementation and depletion has proved beneficial to some COVID-19 patients, opening a debate on the best treatment strategy (Dominic et al., 2021; Grimes et al., 2021). These contradictory findings can be reconciled to some extent as arginine may be both helpful and detrimental depending on the amount of arginase expression. Increased arginase depletes both arginine and NO, predisposing to COVID-19 critical illness and neurodegeneration (Derakhshani et al., 2021; Dean et al., 2021). Therefore, patients with elevated arginase levels, would likely benefit from NO supplementation (but not arginine as this may upregulate the ornithine/urea pathway) (Lotz et al., 2021; Fang et al., 2021). On the other hand, NO and/or arginine supplementation may be detrimental to patients with excessive peroxynitrite as it may upregulate the oxidative stress (Nguyen et al., 2016). For this reason, arginase inhibitors (augment NO and lower peroxynitrite), may be a better therapeutic strategy than either arginine depletion or supplementation (Clemente et al., 2020).

Natural arginase inhibitors, diamino and α-amino acids, as well as flavonoid compounds, such as the plant extract (2S)-5,2′5′-trihydroxy-7,8-dimethoxy flavanone may be beneficial for patients with COVID-19 and neurodegenerative disorders (Girard-Thernier et al., 2015; Minozzo et al., 2018; Clemente et al., 2020; Arraki et al., 2021; Li et al., 2021). Synthetic arginase inhibitors are broad-spectrum anthelmintics, including imidazothiazoles and their derivatives levamisole, oxazolopyridine, azabenzimidazole, found to possess antiviral, anticancer, and anti-AD properties (Al-Horani and Kar, 2020; Weiss et al., 2021). Imidazothiazoles have not been adequately studied but are promising as panviral and neuroprotective agents.

### Calcium Channel Blockers

Recent studies have reported that calcium channel blockers (CCBs) can ameliorate COVID-19 pathology, decreasing morbidity and mortality (Straus et al., 2021). Drugs, including amlodipine, nifedipine, nimodipine, memantine were demonstrated efficacious against SARS-CoV-2 virus and AD, indicating a related pathogenesis (Nimmrich and Eckert, 2013; Solaimanzadeh, 2020)

The natural CCB compounds, bisbenzylisoquinoline alkaloid, neferine and its analogs liensinine and isoliensinine, inhibit Ca2+ mediated cell-cell fusion, suggesting restoration of host antiviral immunity (Minozzo et al., 2018). Another natural compound, *Artemisia annua* extract, artemisinin, blocks several voltage-gated ion channels, including NMDA, indicating potential antiviral and anti-neurodegenerative properties (Qiao et al., 2007). Indeed, studies in rodents and cultured human neurons found that artemisinin ameliorated neurodegenerative pathology, emphasizing the role of dysfunctional Ca2+ signaling in these conditions (Zhao et al., 2020).

Several studies found that muscarinic acetylcholine receptors (mAChR) antagonists lower cytosolic Ca2+, averting the conversion of trophic into neurotoxic astrocytes, suggesting a role in neurodegenerative disorders (Takata et al., 2011). Indeed, M1 and M3 muscarinic receptor antagonists were shown to reverse the cocaine-induced astrocytic neurotoxicity, emphasizing their neuroprotective effects (Garcia et al., 2015; Calcutt et al., 2017).

### TMEM16F Inhibitors

TMEM16F inhibitors, an important class of cell-cell fusion blockers, include a variety of agents, ranging from anthelmintic drugs to psychotropics and anticancer compounds. Recent studies have suggested that viruses and malignancies invade human cells *via* similar pathways, emphasizing that antiviral and anticancer drugs are related. For example, Ivermectin, a macrolide anthelmintic with antiviral properties is also an effective tumor suppressor, suggesting similar action mechanisms (Formiga et al., 2021; Tang et al., 2021) (**Table 3**).

**Table 3:** Potential anti-fusion therapeutic strategies.

COMPOUND	REFERENCES
Natural arginase inhibitors	
diamino acids	(Tirumuru et al., 2016)
α-amino acids S Clemente G	(Tirumuru et al., 2016)
2S)-5,2′5′-trihydroxy-7,8-dimethoxy flavanone	(Tirumuru et al., 2016)
Synthetic arginase inhibitors	
Imidazothiazoles: levamisole, oxazolopyridine, azabenzimidazole	(Liu et al., 2021; Jiang et al., 2021)
**Calcium channel blockers**	
amlodipine, nifedipine, nimodipine, memantine	(Del Papa et al., 2021; Jeon and Jung, 2022)
bisbenzylisoquinoline alkaloid	(Brown and Fisher, 2021)
neferine, liensinine, isoliensinine	(Brown and Fisher, 2021)
artemisinin	(Dörnen et al., 2020)
**TMEM16F inhibitors**	
Ivermectin	(Rems et al., 2013; Zhou et al., 2016; Greenfield, 2018)
Niclosamide, nitazoxanide, hexachlorophene and dichlorophen	(Hou et al., 2006)
Trifluoperazine	(Hou et al., 2006)
serotonin reuptake inhibitors (SSRIs)	(Hou et al., 2006)
epigallocatechin gallate	(Lotz et al., 2021)
**Senolytic drugs**	
hydroxychloroquine	(Minozzo et al., 2018)
azithromycin, minocycline and roxithromycin	(Al-Horani and Kar, 2020; Weiss et al., 2021)
quercetin	(Minozzo et al., 2018)
senolytic vaccine	(Formiga et al., 2021)
antibody–drug conjugates	(Tang et al., 2021)
**Microtubule stabilizing agents**	
TPI-287 (discontinued)	(Malashkevich et al., 2010)
Davunetide (discontinued)	(Malashkevich et al., 2010)
CNDR-51549	(Li et al., 2020)
CNDR-51555	(Li et al., 2020)
CNDR-51657	(Du et al., 2012)
Sabizabulin	(Millington-Burgess and Harper, 2021)
Taccalonolides	(Ousingsawat et al., 2018)
Lithium	(Sargiacomo et al., 2020)

The COVID-19 pandemic drew attention to the connection between pathogens, cancer, and neuropsychiatric disorders, suggesting the possibility of common treatment strategies (Xu et al., 2020). For example, TMEM16F inhibitor niclosamide and its analogs nitazoxanide, hexachlorophene and dichlorophen, present with intriguing anthelmintic, anticancer, and anti-ALS properties, indicating similar pathogenesis (Peng et al., 2021). Indeed, as these agents target S100A4, a protein involved in schizophrenia and inhibited by the phenothiazine class of antipsychotic drugs, a common pathogenetic mechanism is being highlighted (Malashkevich et al., 2010; D’Ambrosi et al., 2021). In addition, since S100A4 has also been implicated in tumorigenesis, it may be the common denominator between viral illness, cancer, and neuropsychiatric disorders (Fei et al., 2017).

Recent *in silico* studies, have shown that several psychotropic drugs, including trifluoperazine and serotonin reuptake inhibitors (SSRIs) block TMEM-16F, explaining their antiviral and anti-syncytial properties (Cavaliere et al., 2019). This is significant, as SSRIs were demonstrated to delay the conversion of mild cognitive impairment (MCI) to AD, further connecting TMEM-16F to neurodegenerative disorders (Bartels et al., 2018).

Natural TMEM16F inhibitors, including the polyphenol Epigallocatechin gallate, have antiviral, anti-neurodegenerative, and anti-cancer properties, emphasizing once more a common action mechanism (Du et al., 2012; Li et al., 2020; Millington-Burgess and Harper, 2021). Another polyphenol, tannic acid, may or may not downregulate TMEM16F as two different studies found conflicting results, indicating that more research is needed in this area (Ousingsawat et al., 2018; Le et al., 2020).

### Senolytic Drugs

Several senolytic drugs with established antiviral properties, including hydroxychloroquine and related agents, lower β- galactosidase, a well-known senescence marker, indicating efficacy against virus-induced senescence (Van Gool et al., 2001). Interestingly, large observational studies showed that hydroxychloroquine may ameliorate AD symptoms, suggesting that senescent cell clearance may be a useful strategy against neurodegenerative disorders (Sargiacomo et al., 2020; Lai et al., 2021). Other senolytic agents with antiviral properties, such as azithromycin, minocycline and roxithromycin, were deemed salutary to COVID-19 patients as they selectively eliminate senescent and virus-infected cells (Forloni et al., 2001; Ozsvari et al., 2018; Osorio et al., 2019). Indeed, several senolytic antibiotics, including tetracyclines, have demonstrated anti-neurodegenerative properties in preclinical studies, emphasizing the link between viruses and neurodegeneration (Diomede et al., 2010; Di Pierro et al., 2021).

The natural senolytic agent, quercetin, an effective antiviral and anti-neurodegeneration compound, is currently in clinical trials for COVID-19 (Khan et al., 2019; Islam et al., 2021)(NCT05037240). Quercetin was found to preempt the development neuronal damage as well as to possess anticancer and anti-inflammatory properties (Vafadar et al., 2020).

A novel senolytic vaccine, recently tested in progeroid mice, may usher a new era in senolytic interventions as it opens the possibility of preventing the development of neurodegenerative disorders, viral infections and possibly cancer (Yoshida et al., 2020; Suda et al., 2021). Furthermore, an antibody–drug conjugate against a membrane senescence marker was demonstrated to clear senescent and virus-infected cells, emphasizing a new senolytic strategy (Poblocka et al., 2021).

### Microtubule Stabilizing Agents

MSAs are compounds that attach to the microtubules, preventing their disassembly. Most drugs targeting microtubules are anticancer agents that may also possess anti-neurodegenerative and antiviral effects (Hung and Fu, 2017; Fernandez-Valenzuela et al., 2020; Tsai et al., 2020; Sirakanyan et al., 2021). Many of these drugs demonstrated beneficial effects in animal models, however those tested in humans are few and include TPI-287 and NAP (Davunetide CP201), an intranasal neuropeptide (NAPVSIPQ)(NAP) (NCT01966666) (Gozes, 2020). These MSAs have not reached the clinic, however, activity-dependent neuroprotective protein (ADNP), derived from NAP, remains a potential hope and is scheduled for future clinical trials (Varidaki et al., 2018; Al-Horani and Kar, 2020; Santiago-Mujika et al., 2021). A recent addition to MSAs, sabizabulin, is currently in clinical trials as an antiviral drug, suggesting possible benefit in tauopathies (NCT04388826) (Malebari et al., 2021).

Natural MSA compounds, CNDR-51549 and CNDR51555 (US patent: US20170173016 A1) were found to cross the blood brain barrier, indicating potential benefit in tauopathies (Kovalevich et al., 2016). Another compound, CNDR-51657, was demonstrated to downregulate the hyperphosphorylated Tau, suggesting a preventive potential (Zhang et al., 2018). Another natural MSA compound and *Tacca* extract, taccalonolides, may benefit AD patients by augmenting tubulin polymerization, reversing the effect of pTau (Murru et al., 2020; Chen et al., 2021). Interestingly, studies from the 1970s observed that lithium was a MSA, raising interesting questions about its established antiviral and neuroprotective properties (Matsunaga et al., 2015; Murru et al., 2020; Chen et al., 2021). Indeed, lithium reverses pTau-induced astrocytic senescence and enhances T-cell function, suggesting senolytic properties (Bhattacharyya and Wolff, 1976; Kucharz et al., 1988; Olson et al., 2019; Viel et al., 2020). This is significant, as lithium can reverse the virus-induced damage of tubulin, a key molecule in T cell activation (Kopf and Kiermaier, 2021).

Taken together, MSAs, many of which are plant extracts, are interesting compounds that require further research as antiviral and neuroprotective agents (Garcia-Montojo and Nath, 2021).

## Conclusions

Viruses augment infectivity by fusing host cells into multinucleated hybrid entities that engender cellular senescence, immunosuppression or immune exhaustion that may predispose to neurodegenerative disorders. The study of physiological and pathological syncytia has emphasized the role of arginine, calcium signaling, TMEM16F and the cytoskeleton in synaptic plasticity, memory, and cognition. These novel findings are likely to contribute to the development of new therapeutic strategies not only for neuropsychiatric conditions but also for cancer and viral infections.

A better understanding of physiological fusogens, their properties and function, will lead to more focused interventions against autoimmune and placental disorders. Adequate HERV inhibition will likely improve the outcome of antiviral and antitumor drugs, opening the possibility of neurodegenerative disorders prevention. This is illustrated by the novel recombinant anti-HERV-W ENV antibody (GNbAC1), currently in clinical trials for MS, and a promising antiviral agent (NCT01639300) (Diebold and Derfuss, 2019; Singh and Bharara Singh, 2020). The same may be true of arginase inhibitors, MSA, and TMEM16F inhibitors, drugs with multiple therapeutic targets.

At present, the study of cell-cell fusion is in its infancy therefore, a better understanding of the molecular underpinnings of syncytia formation would shed light on cellular uptake of pathogens and oncogenes, opening novel avenues for preventive care.

## Author Contributions

All authors listed have made a substantial, direct, and intellectual contribution to the work, and approved it for publication.

## Conflict of Interest

The authors declare that the research was conducted in the absence of any commercial or financial relationships that could be construed as a potential conflict of interest.

## Publisher’s Note

All claims expressed in this article are solely those of the authors and do not necessarily represent those of their affiliated organizations, or those of the publisher, the editors and the reviewers. Any product that may be evaluated in this article, or claim that may be made by its manufacturer, is not guaranteed or endorsed by the publisher.
